# The Protective Effects of a Synthetic Geranyl Acetophenone in a Cellular Model of TNF-α-Induced Pulmonary Epithelial Barrier Dysfunction

**DOI:** 10.3390/molecules23061355

**Published:** 2018-06-05

**Authors:** Tee Yee Sim, Hanis Hazeera Harith, Chau Ling Tham, Nur Fariesha Md Hashim, Khozirah Shaari, Mohd Roslan Sulaiman, Daud Ahmad Israf

**Affiliations:** 1 Department of Biomedical Science, Faculty of Medicine & Health Sciences, Universiti Putra Malaysia, 43400 Serdang, Selangor, Malaysia; racheltysim@gmail.com (T.Y.S.); chauling@upm.edu.my (C.L.T.); nurfariesha@upm.edu.my (N.F.M.H.); mrs@upm.edu.my (M.R.S.); 2 Natural Products Laboratory, Institute of Bioscience, Universiti Putra Malaysia, 43400 Serdang, Selangor, Malaysia; khozirah@upm.edu.my

**Keywords:** acetophenone, acute lung injury, inflammation, alveolar epithelial cells, barrier function, barrier integrity junctional, complex molecules

## Abstract

Alveolar epithelial barrier dysfunction contributes to lung edema and can lead to acute lung injury (ALI). The features include increased epithelial permeability, upregulation of inflammatory mediators and downregulation of junctional complex molecules; these changes are often induced by inflammation. tHGA is an acetophenone analogue with therapeutic potential in asthma. Its therapeutic potential in ALI is presently unknown. Herein, the effects of tHGA on epithelial barrier dysfunction were determined in TNF-α-induced human alveolar epithelial cells. The anti-inflammatory properties of tHGA were assessed by monocyte adhesion assay and analysis of MCP-1 and ICAM-1 expression. The epithelial barrier function was assessed by paracellular permeability and transepithelial electrical resistance (TEER) assays, and analysis of junctional complex molecules expression. To elucidate the mechanism of action, the effects of tHGA on the NF-κB and MAPK pathways were determined. Gene and protein expression were analyzed by RT-PCR and Western blotting or ELISA, respectively. tHGA suppressed leukocyte adhesion to TNF-α-induced epithelium and reduced MCP-1 and ICAM-1 gene expression and secretion. tHGA also increased TEER readings, reduced epithelial permeability and enhanced expression of junctional complex molecules (zona occludens-1, occludin and E-cadherin) in TNF-α-induced cells. Correspondingly, the NF-κB, ERK and p38 MAPK pathways were also inhibited by tHGA. These findings suggest that tHGA is able to preserve alveolar epithelial barrier function in response to acute inflammation, via its anti-inflammatory activity and stabilization of epithelial barrier integrity, mediated by NF-κB, ERK and p38 MAPK signaling.

## 1. Introduction

The alveolar epithelium is a component that forms the blood-air barrier in the lung which is important for gas exchange. The breakdown of alveolar epithelial barrier integrity can lead to acute lung injury (ALI) and acute respiratory distress syndrome (ARDS), with a greater risk in patients with lung infection or chronic inflammatory airway diseases [1,2]. These life-threatening pulmonary disorders are driven by acute inflammation and subsequent accumulation of inflammatory cells and protein-rich fluid in the alveolar space, forming alveolar edema. Inflammatory mediators are known to increase alveolar epithelium permeability, the key event leading to edema, which is regulated by apical junctional complexes [3,4,5]. Within the apical junctional complex, tight junctions are primarily responsible for the control of paracellular transport, whereas adherens junctions are largely responsible for cell-cell adhesion [4,5]. Some components of the apical junctional complex also play significant roles in edema clearance which are important for the resolution of these conditions. The disruption of epithelial barrier integrity by inflammatory mediators is also associated with upregulation of chemokines and adhesion molecules in alveolar epithelial cells, central to inflammatory cells recruitment and infiltration [3,6]. While the inflammatory aspect is an attractive therapeutic target, this alone may not be sufficient to resolve these conditions efficiently. In fact, recent evidence suggests that better outcomes can be achieved with the restoration of structural and functional barrier integrity [7,8].

2,4,6-trihydroxy-3-geranyl acetophenone (tHGA) is an acetophenone analogue initially found in the leaves of Melicope ptelefolia. Repeated chromatographic separation yielded a yellowish amorphous solid compound with molecular formula C_18_H_24_O_4_. It is a form of geranyl acetophenone with simple aromatic ketones which are used as a raw material in the pharmaceutical industry [9]. Several in vitro and in vivo studies have established its anti-inflammatory activity in a range of cellular and disease models [10,11,12,13,14,15,16,17]. In particular, tHGA displays therapeutic potential for the treatment of asthma, a chronic inflammatory airway disease, owing to its anti-inflammatory activity and ability to attenuate airway remodeling [12,14,17]. Recently, tHGA demonstrated protective effects against vascular endothelial barrier dysfunction, suggesting its therapeutic potential in treating vascular inflammatory disorders [13]. This led us to question whether tHGA may exhibit similar protective effects against alveolar epithelial barrier dysfunction, an important event in ALI/ARDS. Thus, the objective of this study is to determine the effects of tHGA on alveolar epithelial barrier function following exposure to the inflammatory cytokine tumour necrosis factor α (TNF-α), and to elucidate its mechanism of action. The present study demonstrates that tHGA exerts protective effects against alveolar epithelial barrier dysfunction in response to acute inflammation, via both its anti-inflammatory activity and ability to stabilize epithelial permeability, as well as the expression of tight and adherens junctions. These protective effects of tHGA on alveolar epithelial barrier function are mediated by its inhibitory effects on the nuclear factor kappa B (NF-κB) pathway and mitogen-activated protein kinase (MAPK) pathway, mainly through the inhibition of p38 and ERK phosphorylation. These findings provide early evidence for the therapeutic potential of tHGA in the treatment of ALI/ARDS.

## 2. Results

### 2.1. Cytotoxicity

**Figure 1:**
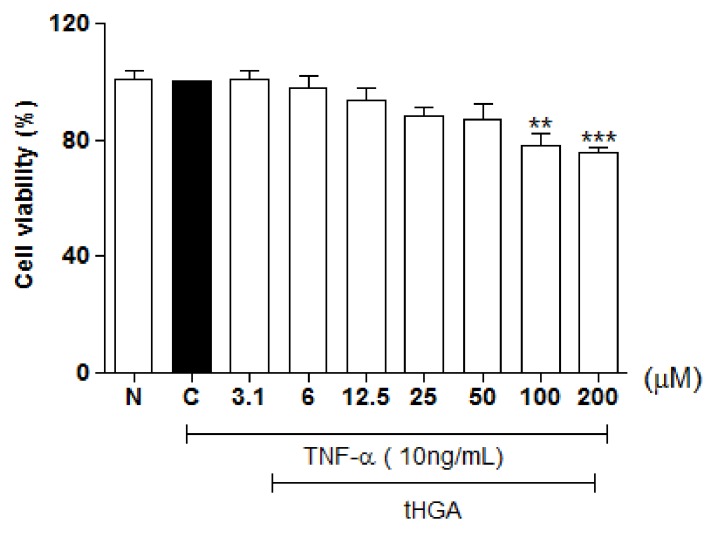
The effect of tHGA on the viability of human alveolar epithelial cells. A549 cells were stimulated with 10 ng/mL of TNF-α and treated with increasing concentrations of tHGA for 24 h, followed by assessment of cell viability by MTT assay. The values are expressed as mean ± SEM of three independent experiments performed in triplicates, where ** *p* ≤ 0.01, *** *p* ≤ 0.001 versus TNF-α-induced cells, according to post hoc comparison using Dunnett’s test. C: TNF-α-induced cells; N: normal and non-induced cells.

Figure 1 shows the effect of tHGA on A549 cell viability. Treatment with tHGA caused a reduction in cell viability at 200 μM (*p* ≤ 0.001) and 100 μM (*p* ≤ 0.01). Therefore, tHGA was used at 50 μM and below for further assays.

### 2.2. tHGA Inhibited TNF-α-Induced Monocyte Adhesion and Transepithelial Migration (TEM)

**Figure 2:**
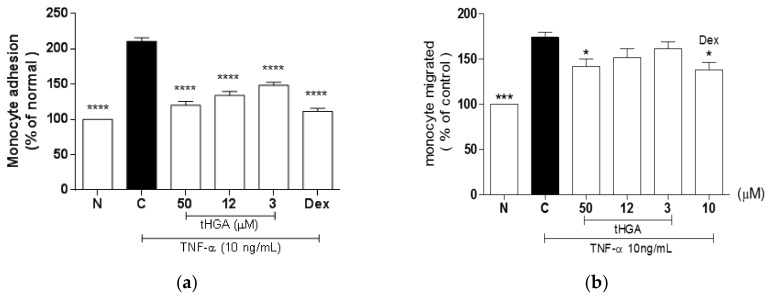
The effects of tHGA on (**a**) monocyte adhesion to and (**b**) migration through A549 monolayer following TNF-α induction. A549 cells were stimulated with 10 ng/mL TNF-α and treated with increasing concentrations of tHGA or positive control for 5 h. For monocyte adhesion assay, A549 were co-incubated with fluorescent-labeled U937 cells for 1 hour. Monocyte adhesion to A549 was presented as the percentage of U937 cells bound to TNF-α induced control group, as described in Methods. For monocyte migration assay, A549 cells were co-incubated with TNF-α activated U937 monocytic cells for 4 h at 37 °C to allow migration process. Monocyte migration was quantified by MTS colorimetric assay. All values are expressed as mean ± SEM of at least three independent experiments, where * *p* ≤ 0.05, *** *p* ≤ 0.001 and **** *p* ≤ 0.0001 versus TNF-α-induced cells, according to post hoc comparison using Dunnett’s test. C: TNF-α-induced cells; N: normal and noninduced cells; Dex: Dexamethasone.

Alveolar edema is a hallmark of ALI/ARDS, which can be induced by acute inflammation. Edema results from increased monocyte adhesion and transepithelial migration (TEM) through epithelial monolayers. The pro-inflammatory mediator TNF-α has been shown to promote monocyte adhesion and TEM in the A549 monolayer [18,19,20]. Hence, we attempted to determine the inhibitory effects of tHGA on the adhesion of U937 cells to A549 cells, and the ability of U937 cells to transmigrate across an A549 monolayer following TNF-α induction. TNF-α induction significantly increased monocyte adhesion (*p* ≤ 0.001); however, co-treatment with tHGA significantly inhibited this effect at as low as 3 µM by 45 ± 4.09% (*p* ≤ 0.001) (Figure 2a). The greatest inhibitory effect of tHGA on monocyte adhesion was observed at 50 µM (81 ± 5.77%), which was comparable to that of the positive control, dexamethasone. In contrast, tHGA was able to inhibit TNF-α-induced TEM effectively only at the highest concentration, 50 µM by 36 ± 8.03% (*p* ≤ 0.05) (Figure 2b).

### 2.3. tHGA Inhibited TNF-α Induced MCP-1 and ICAM-1 Expression and Secretion

**Figure 3:**
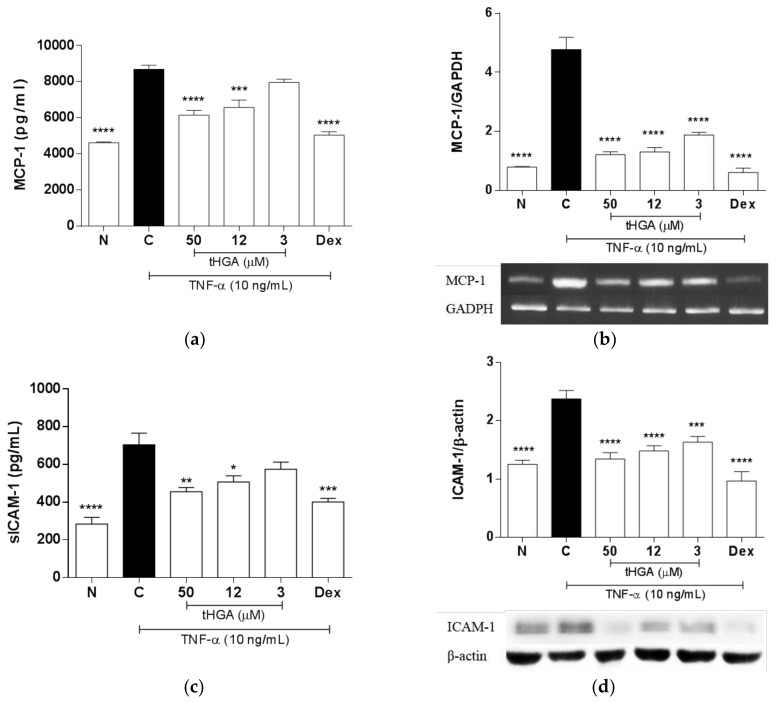
The effects of tHGA on the production of key pro-inflammatory mediators by A549 cells following TNF-α induction. (**a**) The concentration of soluble MCP-1 in A549 cell culture supernatant and (**b**) MCP-1 gene expression in A549 cells following TNF-α induction. (**c**) The concentration of soluble ICAM-1 in A549 cell culture supernatant and (**d**) ICAM-1 protein expression in A549 cells following TNF-α induction. A549 cells were stimulated with 10 ng/mL TNF-α and treated with increasing concentrations of tHGA and positive control for 24 h. The levels of secreted pro-inflammatory mediators were determined by ELISA, as described in Methods. For gene or protein expression analysis, RNA or protein was extracted and subjected to RT-PCR using One Step Reverse Transcriptase-PCR or Western Blotting respectively, as described in Methods. All values are expressed as mean ± SEM of at least three independent experiments, where * *p* ≤ 0.05, ** *p* ≤ 0.01, *** *p* ≤ 0.001 and **** *p* ≤ 0.0001 versus TNF-α-induced cells, according to post hoc comparison using Dunnett’s test. C: TNF-α-induced cells; N: normal and noninduced cells; Dex: Dexamethasone.

TNF-α-induced A549 cells displayed increased TEM in a previous study, with a corresponding increase in chemokines and adhesion molecules expression, including MCP-1 and ICAM-1 [18]. We next examined the effects of tHGA on the expression and secretion of a key chemokine, MCP-1 and an important adhesion molecule, ICAM-1 in TNF-α-induced A549 cells. Figure 3a shows that tHGA significantly reduced TNF-α-induced MCP-1 secretion at 50 µM by 63 ± 2.05% (*p* ≤ 0.0001) and at 12 µM by 38 ± 2.97% (*p* ≤ 0.001). An inhibitory effect was also observed at the gene level whereby tHGA significantly inhibited MCP-1 gene expression at as low as 3 µM (*p* ≤ 0.0001) (Figure 3b). Similarly, tHGA also significantly reduced TNF-α-induced ICAM-1 secretion at 50 µM by 72.5 ± 1.37% (*p* ≤ 0.01) and 12 µM by 60 ± 2.01% (*p* ≤ 0.05) (Figure 3c), as well as ICAM-1 protein expression at all three concentrations by more than 70% (*p* ≤ 0.001) (Figure 3d).

### 2.4. tHGA Reduced Hyperpermeability and Increased Trans-Epithelial Electrical Resistance (TEER) of Epithelium Monolayers

**Figure 4:**
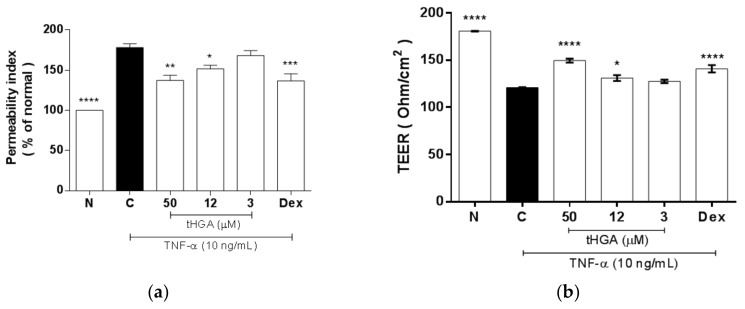
The effects of tHGA on epithelial permeability in response to TNF-α induction in A549 monolayer. (**a**) Fluorescein isothiocyanate (FITC)-dextran fluxes across A549 monolayers. Confluent A549 cells were induced with 10 ng/mL TNF-α and treated with indicated concentrations of tHGA or positive control for 24 h in poly-l-lysine coated cell culture insert. The extent of permeability was measured by fluorescence intensity of the samples collected from basolateral compartment, as described in Methods. (**b**) Transepithelial electrical resistance (TEER) measurement at 3 h post-induction and indicated treatments. A549 cells were seeded onto Transwell inserts and cultured in air-liquid interface for 5 days, followed by induction with 10 ng/mL TNF-α and co-treatment with indicated concentrations of tHGA for 24 h. TEER was monitored for 3 h post induction as described in Methods. All values are expressed as mean ± SEM of at least three independent experiments, where * *p* ≤ 0.05, ** *p* ≤ 0.01, *** *p* ≤ 0.001 and **** *p* ≤ 0.0001 versus TNF-α-induced cells, according to post hoc comparison using Dunnett’s test. C: TNF-α-induced cells; N: normal and noninduced cells; Dex: Dexamethasone.

Apart from cellular changes that promote monocyte adhesion and TEM, edema is also attributed to impaired barrier integrity which is indicated by increased epithelial permeability [3,4]. Changes in the permeability of A549 monolayers to macromolecules and ions were assessed by measuring FITC-dextran flux and TEER, respectively, following 24 h incubation with TNF-α, with or without tHGA or the positive control, dexamethasone. TEER is inversely related to permeability. Therefore, measuring both permeability and TEER can confirm the reproducibility of the experiment. Figure 4a shows that TNF-α significantly increased epithelial permeability (*p* ≤ 0.001), and that tHGA was able to reduce this effect at 50 µM by 50 ± 6.35% (*p* ≤ 0.01) and at 12 µM by 37.5 ± 4.04% (*p* ≤ 0.05). For TEER measurement, TEER values were recorded every 30 min following induction and indicated treatments, for up to 3 h. Figure 4b shows the TEER values for indicated groups at 3 h. Consistent with the permeability assay, TNF-α induction caused a significant reduction on TEER values (*p* ≤ 0.0001), and co-treatment with dexamethasone significantly increased the TEER readings by 47 ± 4.35% (*p* ≤ 0.0001). Co-treatment with 12 µM and 50 µM tHGA significantly increased the TEER readings by 16 ± 2.35% (*p* ≤ 0.05) and 50 ± 4.21% (*p* ≤ 0.0001), respectively.

### 2.5. tHGA Prevented Disruption of Junctional Complexes Molecules

**Figure 5:**
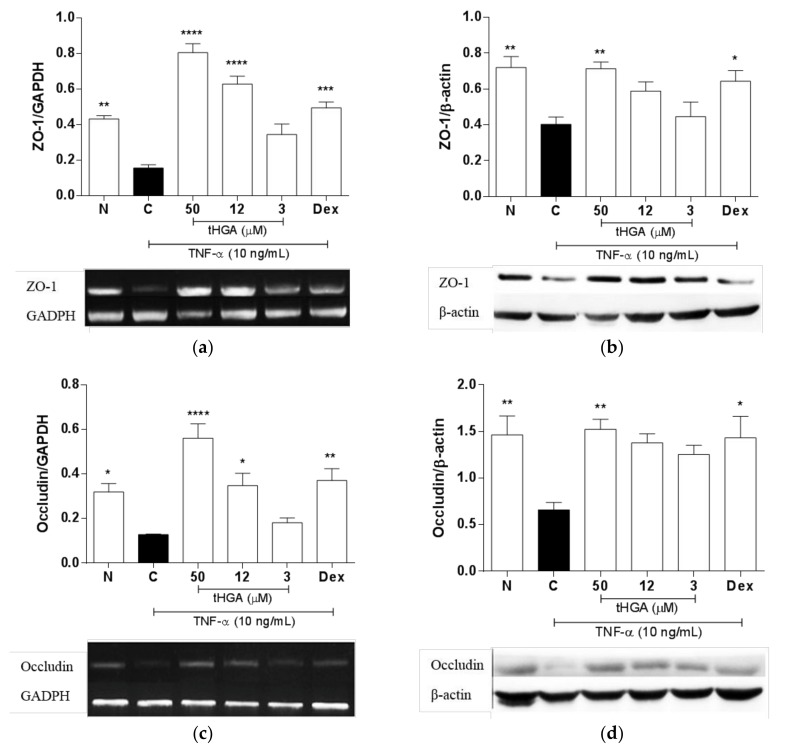

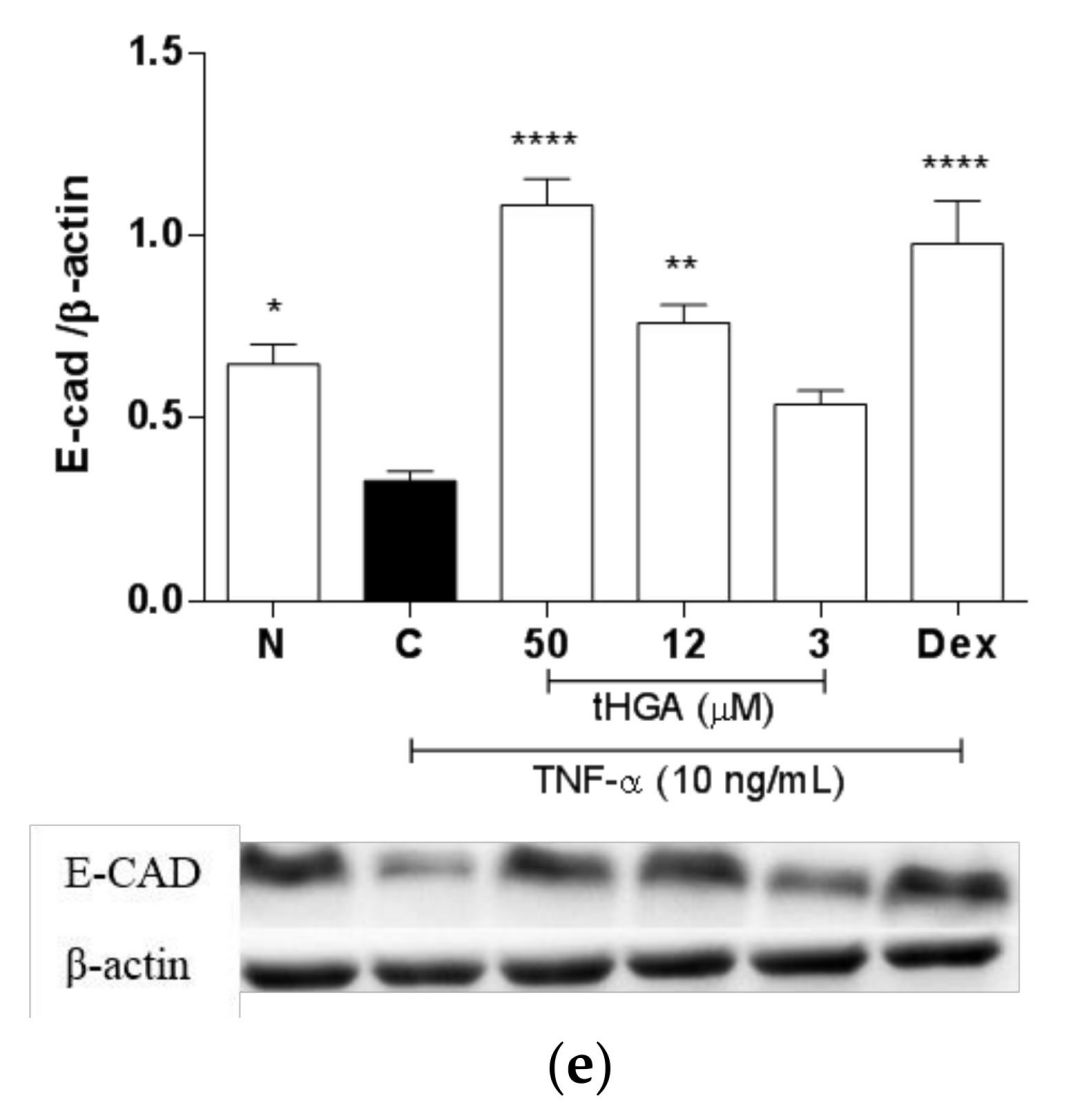
The effects of tHGA on the expression of apical junctional complex molecules in A549 cells following TNF-α induction. (**a**,**b**) The gene and protein expression of tight junctions, ZO-1 and (**c**,**d**) occludin. (**e**) The protein expression of adherens junction E-cadherin in A549 cells following TNF-α induction. A549 cells were stimulated with TNF-α (10 ng/mL) and treated with increasing concentrations of tHGA and positive control for 24 h. RNA or protein was extracted and analyzed using One Step Reverse Transcriptase PCR or Western blot analysis, respectively, as described in Methods. All values are expressed as mean ± SEM of at least three independent experiments, where * *p* ≤ 0.05, ** *p* ≤ 0.01, *** *p* ≤ 0.001 and **** *p* ≤ 0.0001 versus TNF-α-induced cells, according to post hoc comparison using Dunnett’s test. C: TNF-α-induced cells; N: normal and noninduced cells; Dex: Dexamethasone.

Impaired barrier integrity is commonly associated with impaired barrier structure, in which the apical junctional complex plays a major role [4,5]. The apical junctional complex consists of tight junctions which regulate paracellular transport, whereas adherens junctions are mainly responsible for cell-cell adhesion [4,5]. Given that tHGA was able to suppress TNF-α-induced increase in epithelial permeability, whether these effects are associated with changes in the expression of tight junctions, namely zonula occludens-1 (ZO-1) and occludin were examined. Consistent with TNF-α-induced increase in epithelial permeability, TNF-α induction caused significant downregulation of the tight junctions ZO-1 (Figure 5a,b) and occludin (Figure 5c,d) at the gene and protein levels. TNF-α-induced changes on ZO-1 gene expression were reversed significantly by tHGA at 50 µM and 12 µM by 260 ± 6.35% (*p* ≤ 0.0001) and 188 ± 2.15% (*p* ≤ 0.0001), respectively (Figure 5a). Similar to ZO-1, TNF-α-induced changes on occludin gene expression were also reversed significantly by tHGA at 50 µM and 12 µM by 225 ± 2.31% (*p* ≤ 0.0001) and 125 ± 3.10% (*p* ≤ 0.05), respectively (Figure 5c). Of note, 50 µM tHGA not only inhibited TNF-α-induced changes in the tight junctions gene expression, but also appears to further upregulate both ZO-1 and occludin gene expression compared to normal, non-induced levels. However, such an effect was not reflected at the protein level. Furthermore, a significant inhibitory effect of tHGA on the protein expression of ZO-1 (Figure 5b) and occludin was only observed at 50 µM (Figure 5d). Since adherens junctions are also part of the apical junctional complex, the effect of tHGA on the expression of a well-studied adherens junction, E-cadherin, was also assessed. Similar to the tight junctions, E-cadherin protein expression was downregulated by TNF-α, and tHGA was able to reverse this effect at 50 µM and 12 µM by 266 ± 1.22% (*p* ≤ 0.0001) and 133 ± 3.41% (*p* ≤ 0.01), respectively (Figure 5e).

### 2.6. tHGA Inhibited the Activation of NFκB and MAPK Pathways

**Figure 6:**
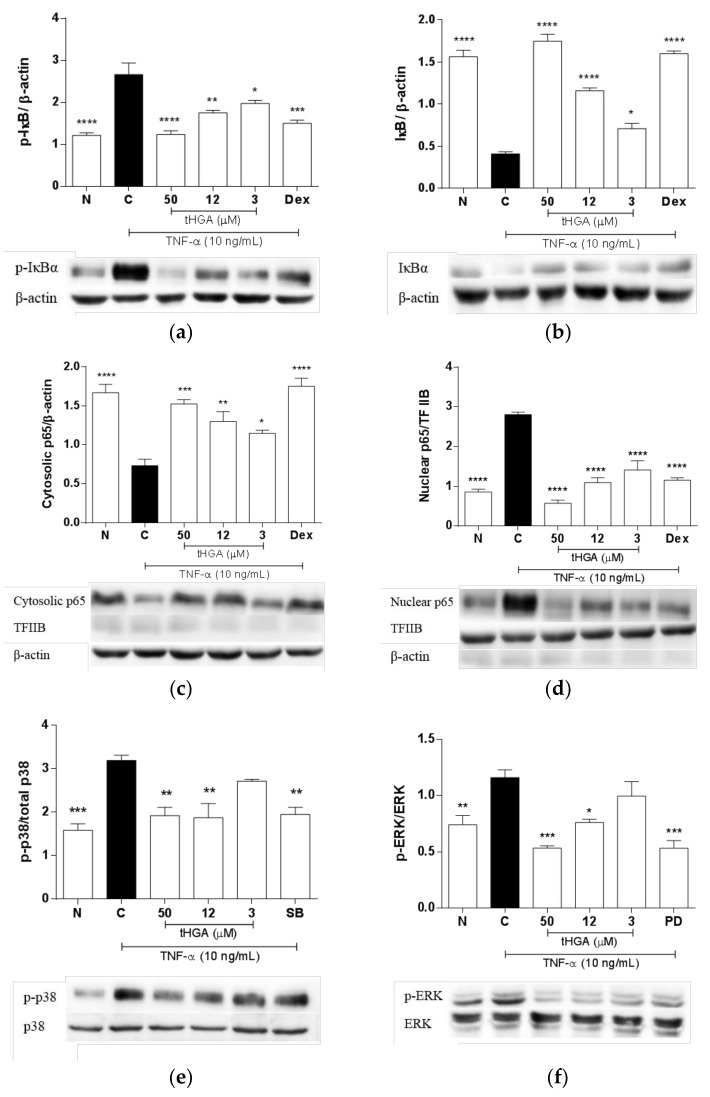
The effects of tHGA on the NFκB and MAPK signaling pathways in A549 cells following TNF-α induction. (**a**) The levels of phosphorylated IκBα, (**b**) total IκBα, (**c**) cytosolic p65 and (**d**) nuclear p65 in A549 cells following TNF-α induction, in the presence of indicated treatments. (**e**) The levels of phosphorylated p38 and (**f**) phosphorylated ERK1/2 in A549 cells following TNF-α induction, in the presence of indicated treatments. A549 were induced with TNF-α (10 ng/mL), with indicated treatments for 60 min. Whole cell extracts were obtained and analyzed by Western blotting. Where necessary, cytosolic and nuclear fractions were obtained prior to Western Blot analysis, as described in Methods. Protein expression were normalized to either β-actin, TFIIB or respective total protein as indicated. Results shown are a representative blot from three independent experiments. All values are expressed as mean ± SEM of three independent experiments performed. * *p* ≤ 0.05, ** *p* ≤ 0.01, *** *p* ≤ 0.001 and **** *p* ≤ 0.0001 versus TNF-α-induced cells, according to post hoc comparison using Dunnett’s test. C: TNF-α-induced cells; N: normal and noninduced cells; Dex: Dexamethasone; SB: SB203580; PD: PD98059.

To determine the inhibitory mechanism of tHGA on TNF-α-induced changes in A549 cells, the effects of tHGA on the NFκB and MAPK signaling pathways were examined. Under normal conditions, NFκB remains inactive due to the binding of inhibitors known as IκB, which block the nuclear localization signals. The activation of NFκB signaling requires IκBα phosphorylation which leads to IκBα degradation; this in turn allows nuclear translocation of NFκB to perform its gene regulatory function [21]. The effect of tHGA on TNF-α-induced activation of the NFκB pathway was determined by assessing the phosphorylation and degradation of IκBα, as well as the nuclear translocation of p65NFκB at 60 min post-induction, with or without tHGA. Dexamethasone was used as the positive control in these experiments. tHGA significantly reduced TNF-α-induced phosphorylation (Figure 6a) and degradation of IκBα (Figure 6b), at as low as 3 µM, by more than 50 ± 2.5% and 25 ± 1.21%, respectively, in a concentration-dependent manner. These also correspond with reduced levels of p65NFκB in cytosolic fractions (Figure 6c) and increased levels of p65NFκB in nuclear fractions (Figure 6d), indicating that tHGA also repressed the nuclear translocation of p65NFκB. For the effects of tHGA on the MAPK pathways, TNF-α-induced phosphorylation of p38 and ERK were assessed at 30 min post-induction. Inhibitors SB203580 and PD98059 were used as the positive controls, as indicated in Figure 6e,f. TNF-α-induced phosphorylation of p38 (Figure 6e) was significantly inhibited by 50 µM tHGA and 12 µM by 94 ± 2.67% (*p* ≤ 0.01) and 82 ± 4.67% (*p* ≤ 0.05), respectively. Meanwhile, phosphorylation of ERK (Figure 6f) was significantly inhibited by 50 µM and 12 µM by 175 ± 2.35% (*p* ≤ 0.01) and 88 ± 4.21% (*p* ≤ 0.05), respectively.

## 3. Discussion

It is accepted that alteration in the lung barrier function in response to injury or infection can lead to lung edema, as a result of inflammation. This state characterizes ALI/ARDS, which, without intervention, has a high mortality rate [1,2]. A balance between the inflammatory response and edema clearance is necessary to resolve this pathological condition [2,3]. Alveolar epithelial cells play a vital role in both these processes, such that alteration in their barrier integrity and function by inflammatory mediators can lead to impaired lung function [3,4]. Alveolar epithelial barrier dysfunction is also attributed to increased expression or secretion of inflammatory mediators, such as chemokines and adhesion molecules by alveolar epithelial cells themselves, which further promote leukocyte adhesion and infiltration, causing edema [3,6]. TNF-α in particular, has been shown to enhance leukocyte adhesion by upregulating chemokines and adhesion molecules expression in alveolar epithelial cells, increasing barrier permeability and downregulating tight junctions expression [18,19,20,22,23,24,25]. The effects of TNF-α on lung epithelial barrier integrity and function are also supported by in vivo studies [26,27,28]. Herein, we report that tHGA is able to suppress TNF-α-induced changes in leukocyte adhesion, epithelial permeability, and the expression of tight junctions. These findings suggest that tHGA exerts beneficial effects on epithelial barrier integrity and function in response to inflammation.

The anti-inflammatory activity of tHGA has been demonstrated by various in vitro and in vivo studies [11,12,13,14,15,16,17]. This is the first report on the anti-inflammatory effects of tHGA in alveolar epithelial cells. Specifically, tHGA effectively suppresses TNF-α-induced monocyte adhesion to alveolar epithelial cells, which is likely to be mediated by its inhibitory effects on MCP-1 and ICAM-1 expression by alveolar epithelial cells. Earlier studies have established that the inhibition of either MCP-1 or ICAM-1 could significantly reduce monocyte transepithelial migration through alveolar epithelial barrier in a TNF-α-dependent manner [18,29]. Interestingly, our findings indicate that the suppression of TNF-α-induced MCP-1 and ICAM-1 expression and secretion by tHGA effectively impedes leukocyte adhesion, but may have limited effects on leukocyte transepithelial migration, particularly at concentrations lower than 50 µM. It is presently unclear why this is the case, as reduced leukocyte adhesion is expected to correlate with reduced leukocyte transmigration [18,19,24,30]. One possible explanation is that there may be other compensatory mechanisms that mediate TNF-α-induced monocyte transepithelial migration, some of which require higher concentration of tHGA to achieve adequate inhibition. Rosseau et al. [18] demonstrated that other epithelial adhesion molecules, such as VCAM-1 and CD47, also play an equally important role as ICAM-1 in mediating TNF-α-induced monocyte migration through A549 monolayers. It is possible that the inhibitory effect of tHGA on ICAM-1 alone is not sufficient to inhibit monocyte transepithelial migration in our experimental setting. Further studies are required to confirm the effects of tHGA on other key molecules involved in TNF-α-induced monocyte transepithelial migration. Nonetheless, such differential effects of tHGA on leukocyte adhesion versus leukocyte transepithelial migration may be useful for the treatment of ALI/ARDS, given that the resolution of ALI/ARDS requires macrophage infiltration in the early stages [2,3]. As such, the administration of tHGA at the appropriate dose and time may allow sufficient leukocyte infiltration for the resolution process, and block excessive leukocyte infiltration to assist in edema clearance. The anti-inflammatory effects of tHGA also appear to be selective and vary in sensitivity, depending on cell type. For instance, tHGA does not inhibit MCP-1 expression, but it inhibits ICAM-1 expression, monocyte adhesion and monocyte migration at as low as 5 µM in vascular endothelial cells [13]. Conversely, a consistent inhibition on MCP-1, ICAM-1, monocyte adhesion and migration through alveolar epithelial monolayers was only observed at 50 µM, whilst 3 µM tHGA did not seem to produce any desirable effects. Variation in its activity and sensitivity emphasizes the importance of assessing tHGA’s activity and mechanism of action in different cellular and animal disease models, to determine whether tHGA may be a suitable option for the treatment of related inflammatory diseases.

Apart from the pro-inflammatory cellular changes, alveolar edema is also attributed to impaired epithelial barrier structure and function [3,4]. The significance of tight junctions, namely ZO-1 and occludin in pulmonary epithelial barrier function, is generally well-established [31,32,33,34]. Specifically, reduced expression of either molecule is associated with impaired alveolar epithelial barrier function in vitro, as indicated by increased permeability or reduced TEER [35,36,37,38,39]. We demonstrate that tHGA can suppress alterations in paracellular permeability and TEER in response to TNF-α, and that this is associated with the restoration of both ZO-1 and occludin expression. Notably, the effects of tHGA on the tight junctions are more prominent at the gene level, compared to the protein expression. In fact, the effects of the highest concentration of tHGA (50 µM) exceeded the normal ZO-1 and occludin gene expression levels, effects which are not reflected at the protein level. The discrepancy in the observed gene and protein expression may be influenced by factors such as modulation of protein’s translation rates, half-life, synthesis, and transport [40,41,42,43,44]. Nevertheless, the highest concentration of tHGA (50 µM) consistently showed significant effects on the gene or protein expression of the tight junctions, and corresponds with improvement in alveolar epithelial barrier function in this study. It is worth noting that, at least in our experimental settings, the excessive induction of tight junctions gene expression by 50 µM tHGA was not associated with negative effects on the epithelial barrier integrity and function. The loss of ZO-1 and occludin is commonly reported in mouse models of ALI, suggesting their importance in maintaining normal lung function [45,46,47,48]. In agreement with our findings, recent evidence supports that the stabilization of ZO-1 and occludin expression could relieve pulmonary edema, as a result of enhanced pulmonary barrier function in a mouse model of ALI [46,49].

The assembly of the tight junctions in epithelial cells involves interaction with adherens junctions [4,5]. In contrast with the tight junctions, evidence on the role of adherens junctions in the regulation of alveolar epithelial barrier function is currently lacking. E-cadherin is one of the most widely studied adherens junctions. Nathalie et al. [50] and Hardyman et al. [32] previously reported a downregulation of E-cadherin expression by TNF-α in human bronchial epithelial cells. The latter study further demonstrated in vitro that reduced E-cadherin expression is associated with barrier dysfunction [32]. Of note, a study reported that the silencing of E-cadherin in bronchial epithelial cells leads to ZO-1 downregulation and impaired structural barrier integrity [51], while another study reported a loss of E-cadherin, ZO-1, and occludin expression in the airway epithelium, following an intratracheal allergen challenge in an asthmatic mouse model [52]. Herein, we demonstrate that, similar to the tight junctions, TNF-α also downregulates E-cadherin expression in alveolar epithelial cells, and that this alteration was inhibited by tHGA. This observation suggests that the loss of E-cadherin may also contribute to impaired alveolar epithelial barrier function in response to acute inflammation. Although the effects of the highest concentration of tHGA (50 µM) exceeded the normal levels of E-cadherin protein expression, this was not associated with unwanted changes in the epithelial barrier function in our experimental settings. Despite this, further investigation is required to examine whether tHGA further induces E-cadherin expression or other junctional complex molecules in other experimental systems or disease models, and whether such a change is associated with undesirable cellular responses. Taken together, it is reasonable to speculate that tHGA’s ability to restore E-cadherin expression may indirectly contribute to the stabilization of alveolar epithelial barrier integrity by facilitating the assembly of occludin and ZO-1 in response to insults. The loss of E-cadherin expression in alveolar epithelial cells has recently been associated with impaired barrier integrity and function in vitro, as indicated by reduced TEER and paracelullar permeability; these effects were also reflected in vivo, as indicated by increased pulmonary inflammation and edema [53]. 

TNF-α is known to activate two major signaling pathways, namely the NFκB and MAPK pathways. Both these pathways are widely recognized to mediate inflammation in various cell types and pathological conditions [21,54], and are also common putative targets for the treatment of conditions involving pulmonary inflammation including ALI/ARDS [55,56,57,58]. A similar mechanism of action has also been reported in tHGA-treated activated mast cells, which is associated with its anti-allergic effect via suppression of inflammatory mediators production [15,16]. In this study, tHGA inhibited the IκBα phosphorylation and p65 nuclear translocation in a concentration-dependent manner, which corresponds with reduced MCP-1 and ICAM-1 production. The pro-inflammatory role of NFκB in activated alveolar epithelial cells is well-documented; NFκB is known to positively regulate the expression of inflammatory mediators and adhesion molecules including MCP-1 and ICAM-1 [19,20,22,23,24,59]. In contrast, the inhibitory effect of tHGA on the MAPK pathway is more complex. MAPK are a family of serine-threonine kinases which control a variety of cellular activities in response to extracellular stimuli, mainly by regulating the expression of associated genes [60]. Herein, we demonstrate that tHGA inhibited TNF-α-induced ERK phosphorylation in a concentration-dependent manner in alveolar epithelial cells. Conversely, both 50 and 12 µM tHGA exerted a comparable inhibitory effect on p38 phosphorylation. In comparison to ERK and p38, whose activation were inhibited by more than 80% compared to control at 50 µM tHGA, JNK activation was only inhibited by approximately 50%, with no significant effects at lower tHGA concentrations (data not shown). These observations indicate that the effects of tHGA on different MAPK signaling cascades activated in TNF-α-induced alveolar epithelial cells may vary in sensitivity, depending on tHGA concentration. Our findings suggest that JNK is less likely to mediate the early inflammatory response in TNF-α-induced alveolar epithelial cells, compared to ERK and p38 MAPK pathway. This is in agreement with reports which show that inhibition or inactivation of the ERK and p38, but not JNK pathway in alveolar epithelial cells, correlates with the downregulation of inflammatory mediators and adhesion molecules, including MCP-1 and ICAM-1 [30,61]. The inflammatory response in alveolar epithelial cells is thought to be predominantly mediated by the NFκB pathway [59]; however, inhibition of ERK or p38 activation in animal models of ALI commonly resulted in reduced inflammatory response, which implies that the MAPK signaling also largely contributes to the pathological process [58,62,63,64]. Some studies suggest a crosstalk between the NFκB and either ERK or p38 signaling cascades in mediating inflammatory response underlying ALI, in which inhibition of either MAPK cascade alleviated LPS-induced lung injury via suppression of NFκB activity [63,64]. Likewise, tHGA displays an inhibitory effect on NFκB, ERK, or p38 cascades, but whether similar crosstalk between these pathways applies to tHGA is yet to be characterized.

As discussed earlier, tHGA not only exerts anti-inflammatory effects, but also attenuates TNF-α-induced increase in alveolar permeability and downregulation of the tight junctions, ZO-1, and occludin. The ability of tHGA to preserve barrier function may be mediated by NFκB, ERK, and p38 MAPK pathways, partly through the modulation of these tight junctions. This is supported by a handful of studies which demonstrated that inhibition or inactivation of these pathways improved the epithelial barrier function, as indicated by alleviation of lung edema in animal models of ALI [56,58,62,63,64,65]. The role of NFκB and ERK in the disruption of alveolar epithelial permeability is further supported by a recent study involving rat primary alveolar epithelial cells induced by cyclic stretch [66]. In fact, inhibition of ERK activity significantly reduced NFκB-dependent increase in epithelial permeability, suggesting an interaction between the NFκB and ERK MAPK pathways. NFκB activity has also been associated with the downregulation of occludin and ZO-1 expression, implicated in alveolar barrier dysfunction and lung edema [65]. In contrast, it was recently reported that basal NFκB activity is required for the normal function of alveolar epithelial barrier, and that unnecessary inhibition can lead to impaired tight junctions integrity and epithelial barrier dysfunction [67]. Notably, our findings indicate that the inhibitory effect of 50 µM tHGA on NFκB activity, which exerts the greatest anti-inflammatory and barrier preservation effect, did not exceed the normal levels of NFκB activity. In comparison to NFκB, there is currently limited evidence linking the MAPK cascades and the regulation of tight junctions in the context of alveolar barrier function. However, Cohen et al. [68] reported that impaired alveolar barrier function in septic rats is due to ERK-mediated downregulation of several tight junctions, whilst the JNK MAPK pathway is not involved. Furthermore, the ERK pathway has been implicated in the dysregulation of barrier function attributed to reduced expression of ZO-1 and occludin in airway epithelial cells [69]. It is presently unknown whether p38 directly regulates ZO-1 or occludin expression in alveolar epithelial cells. However, studies have indicated that p38 activation mediates barrier dysfunction through the downregulation of ZO-1 and/or occludin in other types of epithelium, including kidney [70,71] and intestinal epithelium [72,73].

Taken together, the present findings suggest that tHGA may be able to enhance the resolution process of ALI via its anti-inflammatory activity and barrier protective effects. The treatment of ALI/ARDS remains a challenge as mechanical ventilation remains the only option shown to improve survival in patients [2]. By contrasts, clinical trials on most pharmacological agents known for their anti-inflammatory properties have not been successful so far, and some with inconsistent outcomes [2,3]. This may be due to the limited ability of such pharmacological agents to stabilize the expression of tight junctions [4]. Of note, our findings suggest that tHGA exhibits both anti-inflammatory and barrier protective effects, and thus, may offer additional benefit for the treatment of ALI/ARDS. In addition, our group has previously reported tHGA’s ability to attenuate asthma in acute and chronic asthmatic mouse models via its anti-inflammatory activity and suppression of airway remodeling [12,17]. Together with the present findings, this suggests that tHGA is likely to benefit asthmatic patients with high risk of ALI/ARDS. Further studies are required to determine whether tHGA administration can improve lung function in animal models and patients with ALI, and whether the stages of the disease may influence the beneficial effects of tHGA.

## 4. Materials and Methods

### 4.1. Reagents

**Figure 7:**
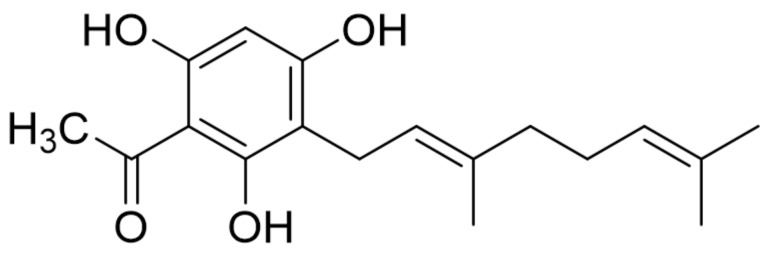
The chemical structure of 2,4,6-trihydroxy-3-geranyl acetophenone (tHGA).

2,4,6-trihydroxy-3-geranyl acetophenone (tHGA) (Figure 7) was synthesized at the Institute of Bioscience, Universiti Putra Malaysia, as described previously [10]. Roswell Park Memorial Institute medium (RPMI)-1640 and Dulbecco’s Modified Eagle Medium (DMEM) were purchased from Hyclone (South Logan, UT, USA). Inter-cellular adhesion molecule 1 (ICAM-1) and monocyte chemotactic protein 1 (MCP-1) immunoassay kits and 0.4 µM, 1 µM and 3 µM pore-sized 24-well cell culture inserts were purchased from R&D Systems (San Diego, CA, USA). Rabbit monoclonal antibody specific for ZO-1 and occludin, and Hoechst 33,342 were purchased from Zymed Laboratories Inc. (San Francisco, CA, USA). Anti-E-cadherin rabbit polyclonal antibody was purchased from Cell Signaling Technology, (Danvers, MA, USA). Rabbit polyclonal antibody specific for ICAM-1, p38, phospho-p38, ERK-1/2, phospho-ERK 1/2, p65NF-κβ, IκBα, phospho-IκBα, TFΠB, and HRP-conjugated donkey anti-rabbit Ig-G and HRP-conjugated mouse anti-β-actin were purchased from Santa Cruz Biotechnology (Santa Cruz, CA, USA). Alexa Fluor 488 was purchased from Abcam (Cambridge, MA, USA). 3-(4,5-dimethylthiazol-2-yl)-2,5-diphenyl tetrazoliumbromide (MTT), bovine serum albumin (BSA) and dimethylsulfoxide (DMSO) were purchased from Amresco (Solon, OH, USA). Polyvinylidene fluoride (PVDF) membrane was purchased from Milipore (Bredford, MA, USA). Super Signal West Femto Maximum Sensitivity Substrate and Bicinchoninic acid (BCA) protein determination kit (Cat No. 23225) were purchased from Milipore (Bredford, IL, USA). One-step RT-PCR kit and RNeasy Extraction kit were purchased from Qiagen (Valencia, CA, USA). Benzonase nuclease, Calcein-AM and MOWIOL 4-88 Reagent were purchased from Calbiochem (San Diego, CA, USA). Gene and protein ladders were purchased from Fermentas (Glen Burnie, MD, USA). Other reagents were purchased from Sigma Chemical Co. (St. Louis, MO, USA).

### 4.2. Cell Culture

A human alveolar epithelial cell line, A549, and a human macrophage-like cell line, U937, were purchased from the American Type Culture Collection (Manassas, VA, USA). A549 and U937 cells were cultured in RPMI-1640 supplemented with 10% FBS, 4.5 g/L glucose, sodium pyruvate (1 mmol/L), l-glutamine (2 mmol/L), streptomycin (50 μg/mL) and penicillin (50 U/mL) and maintained at 37 °C, 5% CO_2_ in a humidified incubator. Cell cultures were split when confluency reached 80–90%. A549 cells were induced with TNF-α for all experiments. DMSO was used as the vehicle for tHGA treatments (0.1% DMSO); therefore, all controls were also treated with 0.1% DMSO, to take into account the effects of the vehicle.

### 4.3. Cell Viability Assay

Cell viability was assessed by the MTT method. In each well of a 96-well plate, 100 µL of cell suspension (1 × 10^4^ cells/well) was loaded, except for blanks. Subsequently, cells were incubated overnight at 37 °C, 5% CO_2_, to allow cells to adhere to wells. After an overnight incubation period, media was gently discarded without disturbing the monolayer of cells. Cells were induced with 10 µg/mL of TNF-α in the presence or absence of treatment at the final volume of 100 µL/well. The tHGA treatment stock was serially diluted in DMSO to decreasing concentrations, ranging from 50 to 3 µmol/L, where the final concentration of DMSO in media was maintained at 0.1%. A total of 100 μL of RPMI-1640 media containing 5% FBS was added to each well, followed by 20 μL of MTT (5 mg/mL). After 4 h, the formazan crystals were dissolved with 100 μL of 100% DMSO per well. The absorbance was measured at λ 570 nm with a microplate reader (UVM 340, ASYS Hitech GmbH, Eugendorf, Austria, Europe), using a reference wavelength of 650 nm. Cell viability was determined as the percentage of untreated stimulated cells.

### 4.4. Monocyte Adhesion Assay

Monocyte adhesion to A549 cells was performed as described previously with modifications [20]. A549 cells were cultured in 96-well tissue culture plates until confluent; TNF-α (10 µg/mL) and increasing concentrations of tHGA or dexamethasone were added concurrently into wells, and incubated for 5 h. A549 cells were then co-cultured with 100 µL of calcium-AM-labeled U937 cells (1 × 10^5^ cells/mL) for 1 h. After non-adhering U937 cells were gently aspirated out, the wells were washed twice with assay buffer (0.5% BSA in DMEM without phenol red), followed by 1X PBS. Adherent U937 cells were lysed with 100 µL of 0.2% Triton X-100. A fluorescence microplate reader (Tecan M200 Infinite, Mannedorf, Switzerland) was used to quantify the fluorescence intensity at 485 nm excitation and 530 nm emission.

### 4.5. Transepithelial Migration (TEM) Assay

The monocyte TEM assay was performed as described previously with modifications [18]. A549 cells (1 × 10^5^ cells/well) were cultured to a well-formed monolayer on poly-l-lysine precoated 24-well plate cell culture insert membranes (3 µM pore size) in RPMI culture medium for 24 h. A549 cells were co-treated with TNF-α (10 µg/mL) and increasing concentrations of tHGA or dexamethasone for 5 h at 37 °C in a 5% CO_2_ incubator. Cell culture inserts were washed with DMEM containing 0.5% BSA, and transferred to a new well which contained 500 µL of DMEM with 20% FBS. TNF-α-activated U937 cells were washed, added into each insert (2 × 10^5^ cells per insert), and incubated for 4 h in 5% CO_2_ at 37 °C. Following incubation, the inserts were discarded and 200 µL of MTS was added into each well, before further incubation for 4 h in 5% CO_2_ at 37 °C. Blank wells contained the same amount of cell culture medium and MTS mixture. Absorbance was measured at 490 nm, with a reference wavelength of 630 nm on a VERSA max microplate reader (Molecular Devices, San Jose, CA, USA).

### 4.6. Cytokine Immunoassay

Spent media from cell viability assay was assayed for concentrations of ICAM-1 and MCP-1 by enzyme immunoassay, according to the manufacturer’s instructions (R&D Systems, Cat No. DY720, DY479 Duo Set).

### 4.7. Reverse-Transcriptase-Polymerase Chain Reaction (RT-PCR)

Total RNA from cell monolayers was isolated using a Qiagen RNeasy Mini Extraction kit (Qiagen, USA, Cat No. 74106), according to manufacturer’s instructions. RNA integrity was examined by formaldehyde agarose gel electrophoresis, and concentrations were determined by UV spectrophotometry (DU 530 Life Science UV/Visible Spectrophotometer, Fullerton, CA, USA). RNA samples were subjected to RT-PCR, using the Qiagen One-Step RT-PCR kit (Qiagen, USA, Cat No. 210212) in an Eppendorf thermal cycler, according to the manufacturer’s instructions. The conditions for the reaction were: 50 °C for 30 min for reverse transcription, 95 °C for 15 min for initial PCR activation, and followed by 72 °C for 10 min for final extension. The reaction products were separated on 1.8% agarose gel and stained with ethidium bromide. PCR products for each gene were determined based on the low molecular weight DNA ladder (New England Biolabs, Ipswich, MA, USA). Primers used were adapted from published human gene sequences (Table 1) [74,75]. Band intensities were quantified by Image J Java-based image processing program (NIH, USA), and were normalized by glyceraldehyde-3-phosphate dehydrogenase (GAPDH) mRNA.

### 4.8. Western Blot Analysis

Protein samples were separated on 10% sodium dodecylsulfate polyacrylamide gel electrophoresis, and transferred onto a PVDF membrane. The membrane was blocked with 5% BSA in Tris Buffered Saline-Tween (TBST) for 2 h at room temperature, and incubated with primary antibody overnight at 4 °C. The primary antibodies used include anti-human ICAM-1 rabbit polyclonal Ab (1:1000 in PBS containing 1% BSA), anti-human E-cadherin rabbit polyclonal Ab (1:1000 in PBS containing 1% BSA), anti-human ZO-1 rabbit mAb (1:250 in PBS containing 1% BSA), anti-human occludin rabbit mAb (1:250 in PBS containing 1% BSA), p38 (1:1000 in PBS containing 1% BSA), p-p38 (1:1000 in PBS containing 1% BSA), ERK (1:1000 in PBS containing 1% BSA), p-ERK (1:1000 in PBS containing 1% BSA), p65NF-κβ (1:1000 in PBS containing 1% BSA), IκBα (1:1000 in PBS containing 1% BSA), p-IκBα (1:1000 in PBS containing 1% BSA, β-actin (1:1000 in PBS containing 1% BSA), and TFIIB (1:1000 in PBS containing 1% BSA). This was followed by the washing step and incubation with horseradish peroxidase-conjugated donkey anti-rabbit secondary antibody (1:5000) for another 2 h at room temperature. Signals were detected using enhanced chemiluminescence (Milipore, Bredford, IL, USA), and imaged in a CCD camera imaging system (Vilber Lourmet, Marne-la-Vallee, France).

### 4.9. Paracellular Permeability Assay

The permeability of cell monolayers was determined by FITC-dextran fluxes across the cell monolayers. A549 cells (2 × 10^4^ cells/well) were cultured on a poly-l-lysine-precoated 24-well plate cell culture insert membrane (1 µM pore size) in RPMI culture medium for 24 h until a well-formed monolayer was observed. Monolayer permeability was disrupted with 10 ng/mL of TNF-α, and co-treated with varying concentrations of tHGA or dexamethasone (10 µM) as positive drug control for 24 h. Treatment medium was carefully removed from each insert and receiver plate well. Following treatment, 500 μL of growth medium was added to each receiver plate well, and 100 µL of FITC-dextran (1 mg/mL) was added to the apical compartment and was allowed to permeate the monolayer for 20 min, protected from light. Inserts were removed from the receiver wells, the medium in the receiver wells was then carefully mixed, and 100 μL was removed from each to a black 96-well opaque plate for fluorescence measurement. Fluorescence intensity was quantified at 485 nm excitation and 530 nm emission (Tecan M200 Infinite, Mannedorf, Switzerland).

### 4.10. Transepithelial Electrical Resistance

A549 cells were seeded onto Transwell inserts (0.4 µM pore size) (BD Pharmingen, San Diego, CA, USA) at a density of 1 × 10^5^ cells/cm^2^. Cells were grown on air-liquid interface (ALI) for 5 days, followed by induction with 10 ng/mL of TNF-α and varying concentrations of tHGA for 24 h. Monolayer integrity was assessed by measuring transepithelial electrical resistance (TEER) with a Millicell-ERS voltmeter (Millipore, Bredford, MA, USA) every 30 min over a 3 h period. TEER (Ohms × cm^2^) was determined as (TEER sample—TEER blank) × surface area (cm^2^). ALI culture was optimized to achieve highest TEER value prior to induction with TNF-α.

### 4.11. Preparation of Whole Cell Lysates and Subcellular Fractions

Following treatments, media was discarded and cells were rinsed twice with ice-cold PBS. Cells were lysed in sample lysis buffer (125 mM, 4% SDS, 20% glycerol, 0.004% bromophenol blue) with phosphatase inhibitor cocktail (Calbiochem, CA, USA). Benzonase nuclease (25 U/mL) was added to digest nucleic acid. Following incubation on ice for 15 min, lysates were boiled at 100 °C for 5 min to denature protein and unfold DNA. Lysates were cooled on ice and centrifuged at 14,000× *g* at 4 °C for 5 min, and the supernatant was collected. Cytosolic and nuclear extracts were obtained using Chemicon’s Nuclear Extraction Kit (Milipore, Bredford, IL, USA), according to the manufacturer’s instructions. Whole cell lysates, cytosolic and nuclear fractions were stored at −80 °C prior to analysis. Protein content was quantified using a BCA assay kit (Pierce, Rockford, IL, USA).

### 4.12. Statistical Analysis

Data is presented as means ± S.E.M from at least three separate experiments. One-way analysis of variance (ANOVA) followed by Dunnett’s post hoc comparisons to analyze the significance of differences between treatments. *p*-values of less than 0.05 were considered significant.

## 5. Conclusions

tHGA alleviates TNF-α-induced alveolar epithelial barrier dysfunction via inhibition of the NF-κB, p38 and ERK MAPK pathways. 

## Figures and Tables

**Table 1:** List of primers used in RT-PCR.

Primer	Sequences (5′-3′)	Product Size (bp)
MCP-1	forward-GCTCATAGCAGCCACCTTCATTC	297
	reverse-TGCAGATTCTTGGGTTGTGGAG	
ZO-1	forward-AGAAGATAGCCCTGCAGC	252
	reverse–AGTCCATAGGGAGATTCC	
Occludin	forward–GATCAGGAATATCCACC	199
	reverse–ATTGTACTCGTCAGCAGC	
GAPDH	forward-GCCATCGACCCCTTCATTGAC	606
	reverse-ACGGAAGGACATGCCAGTGAGCTT	
